# A qualitative study exploring barriers and facilitators in deceased organ donation process among transplant coordinators in India

**DOI:** 10.1038/s41598-024-80290-9

**Published:** 2024-11-20

**Authors:** Britzer Paul Vincent, Gurch Randhawa, Erica Cook

**Affiliations:** 1 Institute for Health Research, Faculty of Health and Social Sciences, University of Bedfordshire, Luton, UK; 2 Department of Psychology, University of Bedfordshire, Luton, UK

**Keywords:** Deceased organ donation, India, Transplant coordinators, Qualitative research, Barriers, Institutional, Health care, Medical research

## Abstract

Although India’s organ donation rate is less than 1 per million population, significant disparities exist between the regions and centres within the country, leading to varying consent rates among different organ donation centres. Therefore, this study aimed to understand the experience of transplant coordinators and their barriers and facilitators in the deceased organ donation process across various organ donation centres in India. A phenomenological study using interviews was conducted among fourteen transplant coordinators purposefully recruited from public and private organ donation centres in India, with experience between six years and more than a decade. Audio recordings were transcribed and analysed using framework analysis. Five themes were identified namely: (1) supportive management policies, (2) infrastructure for the deceased organ donation process, (3) delays in the processing time, (4) active involvement in the identification process, and (5) explaining the concept of brain death. The study identifies that implementation priorities include strengthening teamwork, streamlining processes, optimising infrastructure for sensitive discussions, efficient donor identification, and empathetic handling of donor families’ grief stages. Collaborating with law enforcement, applying successful medico-legal strategies, improving family communication, and clarifying brain death concepts ethically and legally can boost consent rates, fostering informed decisions and possibly achieving self-sufficiency in deceased organ donation.

**Supplementary Information:**

The online version contains supplementary material available at 10.1038/s41598-024-80290-9.

## Introduction

The 63^rd^ World Health Assembly resolution and the third World Health Organisation global consultation on organ donation and transplantation held in Madrid resulted in the ‘Madrid Resolution^1,2^. This called for ‘self-sufficiency’ in each country to reach equitable transplant services and improve deceased organ donations. While taking lessons from other countries to reach ‘self-sufficiency’ in India, it is also essential to learn what experiences or what makes specific centres within the same country contribute efficiently, compared with others. This would help identify any cultural context and work practice experiences that would have helped specific centres/regions perform well compared to others. Identifying such strategies and standardising them across the country could serve as a potential to improve deceased organ donation nationwide.

Deceased organ donation plays a crucial role in the self-sufficiency model, where the goal is to maximise both the number of donors, and the number of organs recovered and transplanted from each donor. However, only living organ donations have constantly been higher than deceased organ donations in India, since its begining^3,4^. In India, between 2013 and 2018, among 49,155 transplants, 10,155 were deceased-donor organ recipients (i.e., 20.66%)^5^. In addition, the National Organ and Tissue Transplant Organisation (NOTTO) in India reported that only 7% of organ transplants were from deceased donors^6^. However, this condition in India is not purely due to the lack of potential deceased donors, but due to the lower rates of eligible deceased organ donors converting to actual donors^7^. This is in line with a Spanish organ donation report that the shortage of organs is not just the lack of suitable organ donors but the failure of the system to convert available potential donors into actual donors^8^ attributing barriers to poor identification, infrastructure, or other barriers within the process of deceased organ donation^9–11^.

While organ donation is an umbrella term for both living and deceased donation, they are different to each other in various aspects. One can argue multidisciplinary infrastructure is common for both living and deceased donations, but infrastructurally the pathway/process of the departments and organisations involved vary significantly, as does the journey of the potential donors and their family members^5^.

In the case of living organ donation, the potential donor is alive and holds the final decision-making power. The potential donor is often concerned about possible health complications that may arise after the donation and lifestyle interference^12,13^. To ensure the donor is fit for the procedure, a comprehensive health and psycho-social assessment is conducted by multidisciplinary team, evaluating the individual’s physical, mental, and legal readiness for donation^14^.

In contrast, for deceased organ donation, in countries with soft opt-in/opt-out policy, family plays the final decision-making role, even if the deceased had expressed/not expressed their wish^15^. And families’ decisions are often influenced by cultural, bodily, and religious perspectives on life after death, which can raise concerns^16,17^. Families also have doubts or anxieties regarding the concept of brain death and the trust with the healthcare^16,18,19^. In such cases, it is crucial for doctors to ensure that families understand the medical realities of brain death as death compared to cardiac death where the heart stops beating, concepts which are not there in the living donation process^18^. Additionally, transplant coordinators have the delicate task of addressing families’ grief in a compassionate and step-by-step manner^20^. They must navigate the emotionally challenging process of introducing the topic of deceased organ donation during such difficult times, ensuring sensitivity and support throughout the conversation^21–24^, which requires professional trainings^10,25^. This shows the different challenges within the process/pathway of deceased organ donation compared to living donation.

In the Spanish organ donation model, the appointment and active involvement of ‘transplant coordinators’ in the identification process are identified as one of the key elements for their higher performances in consent rates^11^. Their involvement includes donor identification, donor evaluation, compliance with medical and legal requirements, and logistic arrangements, while obstacles at any of these would result in losing potential donors^9–11^. As well as networking with various teams involved in the process of organ donation, their relationship with each other, their views on organ donation, and the bereaved families also play an important role^11,26,27^.

The Transplantation of Human Organs and Tissues Act 1994 (as amended in 2011) in India, made the presence of a ‘transplant coordinator’ mandatory in centres for the recovery of organs^28^. Also, several leading stakeholders in transplantation in India have stressed the importance of having ‘transplant coordinators’^18,29^, considering their potential impact on the efficient donation process. Transplant coordinators in India are involved in grief counselling, obtaining written consent, coordinating the process with various stakeholders, maintaining records, psychosocial support, and clarifying the families’ concerns^30–32^. However, in India, there is a gap in understanding the transplant coordinators’ subjectivist experiences who are working in deceased organ donation processes at various centres. Also, an article by Tong, Morton and Webster (2016) argued the necessity of qualitative research in understanding the experiences of stakeholders in transplantation to inform efficient patient-centred practice. The clinical scenario given in that paper is well-aligned with the aim of the present study^33^.

Adding to this gap in knowledge, another leading stakeholder Jha (2014) stressed the importance of conducting qualitative research in identifying the barriers and facilitators toward deceased organ donation programs in India^34^, thereby moving toward self-sufficiency by addressing those identified issues. Therefore, the present study aimed to explore the experiences of transplant coordinators in India leading to an efficient deceased donation process, their barriers, and facilitators.

## Materials and methods

This manuscript follows the consolidated criteria for reporting qualitative research (COREQ): A 32-item checklist for interviews and focus groups^35^ (Supplementary file 1). All methods were performed in accordance to relevant guidelines and regulations, as per the Ethical Approval from the Institute for Health Research Ethics Committee (IHREC952), University of Bedfordshire, United Kingdom.

### Study approach

A qualitative phenomenological design was adopted to explore the lived experiences of transplant coordinators in the work-practice culture they function around^33,36,37^. The phenomenological approach focuses on the conscious reflections, judgement, perception, and emotions of individuals living through a particular experience and what the experience was like to live in it^38^, such as the experience of deceased organ donor family members^39^ and the experiences of transplant coordinators in the consent process^40^. Therefore, in this study, the phenomenon being studied is the lived experiences of transplant coordinators in the process of deceased organ donation in various centres from north and south of India, through which their barriers and facilitators are explored.

### Study setting

Internationally, studies have shown that different work-practice cultures in the organ donation centres could influence the work experiences of the team which will eventually impact outcomes, such as consent rates^5,7,41^. While the organ donation rate in India is less than 1 per million population, the consent rate is highly varied across the centres in the country^1,5,29,42^. Therefore, organ donation centres from northern and southern regions of India were selected for this study. This included areas in and around Chandigarh and Delhi region (i.e., from the northern region of India), and Chennai (i.e., from the southern region of India). These study site regions differed in the number of donors and consent rates, with the southern region performing higher compared to the north^1,7,30^. To address this, stratified purposive sampling was adopted with its first level as ‘*comparative purposive sampling*’ to compare the cases and the second level as ‘*key-informant sampling*’^37^. Some may argue that the southern region in India has been a pioneer in organ donation and that it has a higher number of transplant centres leading to a higher number of donors/transplants, which is true^42^. However, this study does not aim to understand why there is a higher number of donors/transplants in one centre/region compared to others, which is a different concern. But this study specifically explores what happens within the centres that are already approved and undertakes deceased organ donations, their work practices, barriers and facilitators, through the experiences of transplant coordinators. Identifying them can enable centres to standardise the best practices and improve the efficient practice of deceased organ donation.

### Study participants

In India, like the Spanish Organ Donation Model^9–11,26,27^, transplant coordinators are one of the key players involved in the deceased organ donation process from the beginning to the end^18,28–32^. Therefore, to understand the experience of transplant coordinators and address the aim of this study, only those working in the specified regions mentioned above were eligible for participation. The Indian health system is composed of a mixture of public and private healthcare providers, which is even the same in organ donation and transplant services^42^. Therefore, transplant coordinators from public and private transplant centres with more than two years of experience in the deceased organ donation process were included in the study, as this amount of period would have given them hands-on experience, given the donation rate in India. Since India is linguistically diverse and complex^43^, only those who spoke the common or local languages of the study sites were included. These were Tamil and/or English in Chennai, Tamil Nadu and Punjabi, Hindi, and/or English in and around Chandigarh and Delhi were included. However, all of the participants spoke in English. Transplant coordinators who worked only for living and/or tissue donation were excluded from the study as they were not within the scope of the current study.

The researcher (BPV) reached out to several potential participants through various platforms such as the transplant coordinators’ network and a not-for-profit organisation that trains transplant coordinators (MOHAN Foundation). A total of fourteen transplant coordinators took part in the study, seven males, and seven females. Nine were from the southern region and five were from the northern region. Among them, eleven had experience in both public and private hospitals, while the other three had experience only in public hospitals. The sample size was stopped at fourteen participants when the interviews reached data sufficiency and saturation to answer the aim of the study^20,44^.

### Data collection

The interviews were undertaken during the COVID-19 (coronavirus disease of 2019) period. Given the travel restrictions, the researcher (BPV) was not able to travel across the borders, from the United Kingdom. Therefore, telephone interviews with verbal consent were undertaken, a commonly used method when the researcher and the participants are displaced by space and time^45–48^. The interviews were conducted by BPV, who is a male PhD scholar with over three years of work and research experience in public and private transplant centres. Interviews lasted between forty-five and eighty minutes (Table 1). The semi-structured interview guide (Supplementary file 2) was developed based on the role and involvement of transplant coordinators in the deceased organ donation process^30–32^ and piloted. All the interviews were audio-recorded and transcribed for data analysis. To ensure the accuracy of the transcripts, they were also verified randomly by the other two authors.

**Table 1:** Demography of the study participants.

S. No	Participant	Gender	Designation	Region
1	TC1N	Female	Transplant coordinator	North
2	TC2S	Female	Transplant coordinator	South
3	TC3S	Male	Transplant coordinator	South
4	TC4S	Male	Transplant coordinator	South
5	TC5S	Male	Transplant coordinator	South
6	TC6S	Male	Transplant coordinator	South
7	TC7S	Male	Transplant coordinator	South
8	TC8N	Female	Transplant coordinator	North
9	TC9S	Female	Transplant coordinator	South
10	TC10N	Male	Transplant coordinator	North
11	TC11N	Female	Transplant coordinator	North
12	TC12S	Female	Transplant coordinator	South
13	TC13S	Male	Transplant coordinator	South
14	TC14N	Female	Transplant coordinator	North

### Data analysis

Framework analysis^49^ was used to analyse the data as it enabled case-to-case comparison to identify the similarities and differences across and within the study sites sampled. This analysis included familiarising with the transcript, coding, grouping codes into themes, charting data into a matrix, and finally interpreting the data^50^. Three researchers BPV, VS, and ST transcribed the audio recordings, and BPV exported them to NVivo v11 for inductive data coding and analysis. While BPV was involved in all the stages of analysis, GR and EC were involved as second and third reviewers of the analysis right from coding to interpretation.

## Findings

**Fig. 1:**
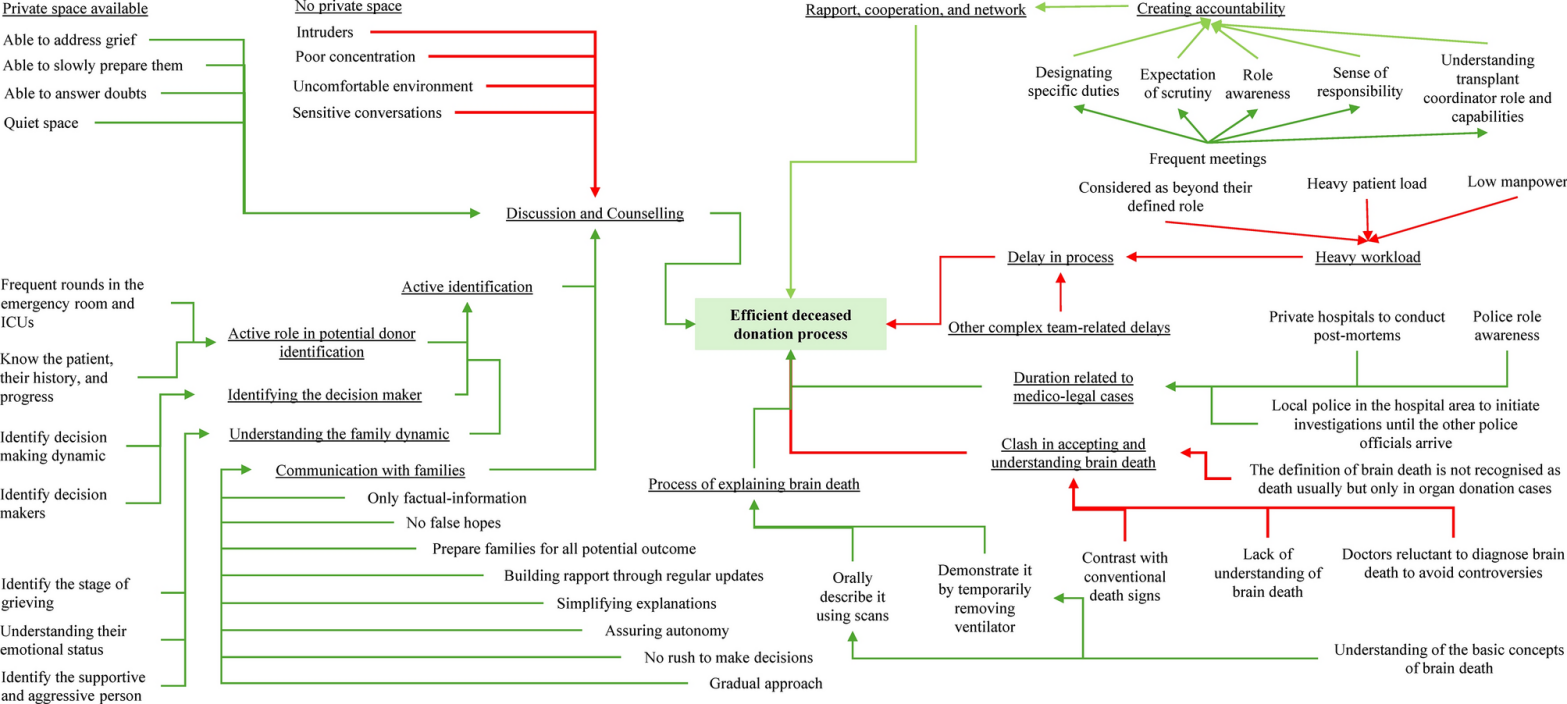
Barriers and facilitators contributing to efficient deceased organ donation process.

From the fourteen interviews, five key themes at institutional level practices were identified inductively: (1) Supportive management policies, (2) Infrastructure for the deceased organ donation process, (3) Delays in the processing time, (4) Active involvement in the identification process, and (5) Explaining the concept of brain death (Table 2 and Fig. 1).

**Table 2:** Sample quotes to support the findings.

Themes	Sub-themes	Quotes
Supportive management policies	Creating accountability	“We need much support from the management, if they create a clear program, telling the roles and responsibilities of each stakeholder… and make them accountable…, it will really work well… we need good management who understands the importance of organ donation and makes each and everyone in the team accountable for their responsibilities.”—**TC9S**
		“In the private sector, there is a designated person for each, and every work and they are accountable for their responsibilities. This makes them work well. Because they are accountable, and they will be questioned if there is trouble in their assigned work. This creates an enabling environment.”—**TC7S**
		“Weekly meetings and updates on these events will re-enforce the stakeholders on the importance their organisation gives to the organ donation process. This is what happens in private hospitals and that is why it is smoother in private hospitals.”—**TC5S**
		“Monthly reporting to government parties regarding several indicators should be made mandatory from the government… only then this program moves forward.”—**TC1N**
	Rapport, cooperation, and network	“You will need to have a proper rapport with the hospital team in all the levels of services, right from senior consultants to housekeeping, especially in government hospitals. This helps you in time of identification, passing on information, getting the lab investigations done and all such things.”—**TC8N**
		“Having good connections with other coordinators, and organ donation organisations will be helpful. This is mainly because we can learn a lot from our own peer colleagues who will help from their experiences.”—**TC6S**
		“We are using a WhatsApp group where the senior faculty members, transfer coordinators, and other residents who are working in the particular wards are in that group. And they have to report immediately whenever there is any one patient or potential brain-dead patient.”—**TC10N**
	Heavy workload	“Though it is a great place to work, the work of a transplant coordinator is a very heavy work job. A transplant coordinator must be working in all the stages right from identification to the time of procurement.”—**TC2S**
		*“In government hospitals, all the work has to be done by the coordinator itself. Even getting blood work to be done we have to keep on chasing it. Whereas, when you look at private hospitals, it is all properly channelised. If I do my work and write my comments, the other person will do it automatically. Things have proper protocol and all work as a team. "—***TC9S**
		“Look, doctors already have a lot of work on their heads, especially within the public hospital patient load is very high. Why will someone take it on their head above their already existing patients, when they are not recognised or given an incentive or any kind of benefits? Why would they want to go in the middle of the night?” –**TC11N**
Infrastructure for the deceased organ donation process	Lack of private space for discussion and counselling	“In government, we do not have proper space to talk with the families. We have to talk to the families in the corridor which is haphazard for such a conversation among the grieving family. It will be crowded, we cannot have this conversation at ease, the family will be standing, and there will be too much noise and interruption. This is a great barrier in government hospitals.”—**TC8N**
		"When I was talking to a bereaved family, in that [public] sort of area, a housekeeping woman came, interrupted my conversation, and in front of the bereaved families asked me if this was a cadaver case. This is such a bad situation in such counselling time. It creates even more pain, suspicion, or mistrust issues among the family members. Sometimes, we would have not even gone to that stage of the conversation, in such times when such interruption happens, it badly influences the counselling.”—**TC9S**
		“In private hospitals, the counselling rooms are immediately next to the ICU and there are beautiful rooms for the families to have this conversation and safe as well because the room has a CCTV.”—**TC14N**
	Lack of proper infrastructure in government hospitals	“The first barrier is the lack of proper infrastructure to maintain the brain death patient. We need proper infrastructure such as a well-equipped ICU and well trained and informed team in organ donation. The other issue is with the perception of doctors working in organ donation centres, their cooperation, and administrative works.”—**TC14N**
Delays in the process time	Delays related to medico-legal cases	“According to the Tamil Nadu state government, we are now allowed to do post-mortem wherever the donation is taking place, be it private also… In that way the family also feel comfortable for organ donation as it avoids delays.”—**TC7S**
		“If they are in a private hospital, a post-mortem will take place in a government hospital delaying the process. At least for donor families, post-mortem should take place in the same hospital.”—**TC8N**
		“The Tamil Nadu government has authorised the local outpost inspector to file the FIR or investigate the case when the police from the accident site are absent. We have tie-ups with the police department, and we give organ donation awareness programs to them and make them feel why their role is very important. This creates more support from the police officers.”—**TC2S**
	Other complex team-related delays	“Many things like the post-mortem, night-time declaration, lack of a 24-h transplant coordinator presence, several blood investigations and all such things delay the process. Therefore, this delay becomes a challenge for the bereaved families”—**TC11N**
Active involvement in the identification process	Active role in potential donor identification	“Everyday morning, we go for rounds in every ICU, this makes us aware of how many cases are critical, how many are head injuries, how many are in ventilator, how many have GCS 3. Then we go for a follow-up round. We just keep a note of what is the status of the patients.”—**TC13S**
		“In rounds, you become aware of the potential donors in the hospital, in such times you will know what cases are there and their criticality.”—**TC12S**
	Understanding the family dynamic	“We would observe the family members, analyse how many people are there, what is their mindset and what kind of family they are. Following this we would then approach the family. We would do all these initial approaches (i.e., analysing the family, and rapport building) before the apnoea test. I feel this approach makes the process easier, more comfortable, and trustable.”—**TC3S**
	Identifying the decision-maker	“I will observe or collect data from the nursing staff who is treating the patient, if the family is able to understand or if the family is accepting the prognosis, the person whom they feel is the decision maker in that family, who’s coming in to ask about the status and who is bringing the necessity items for the patient, who can influence the decision of the family.”—**TC10N**
		“I identify the most influential person in the family, along with the next-of-kin who has the authority to make the decision. In that way, it is easier to get them all on one track. If I miss the most influential family member and then counsel only the next of kin, the family member who has high influence has greater potential to differ.”—**TC8N**
Explaining the concept of brain death	Clash in accepting and understanding brain death	“Firstly, there is no proper understanding of brain death among the public. When a patient is declared brain dead, who looks to have chest movement, a beating heart, and a warm body, there is a high risk that the family may not understand the concept of brain death properly, where the doctor may end up in a lot of controversies and trouble, despite him declaring brain death based on internationally followed guidelines. Therefore, this is one great barrier for doctors to indulge in such activities freely”—**TC14N**
		“I feel that the definition of brain death should be recognised as a death in general death definition by law. Whereas now, brain death is defined as death only in the organ donation law. We are not declaring brain death just for organ donation this is a misconception among the people in our country. Therefore, the fear of man is that doctors are declaring brain death for the sake of organ donation.”—**TC9S**
	Process of explaining	“Usually, the patients will have some spinal reflexes; they should not be misunderstood and thought that the patient is alive. Hence before taking them into to ICU to demonstrate brain death, we should explain all these things, if not it might end up becoming a devastating event not only to the hospital but to the whole concept of organ donation. We should give them certain examples like how the body moves while burning the corpse. Only when they understand the concepts of brain death it is safe to take them to ICU and demonstrate”—**TC3S** “What we often do to help families understand spinal reflexes is to explain it using familiar, local examples. For instance, we might mention the movements observed in a deceased person during cremation or the twitching of a chicken when a butcher removes its head. Then, we ask them whether those movements imply that life still exists. This approach helps bridge the gap between medical concepts and their lived experiences.”—**TC2S**
	Clearly answer their doubts	“We update the family’s stage-by-stage. We will inform them that the patient’s condition is sick, and we may expect adverse outcomes, and prepare them to be ready to face. This gradual approach has helped. However, informing them suddenly will bring a great shock and less time to grieve and come to a state where they can talk about brain death and organ donation.”—**TC4S**
		“The other thing is it is always good they say the facts as they are and not try to console or comfort the family by giving them false hopes. Giving false hope for such reasons will make the donation less likely. The team here informs the family of the true facts and never gives them false hopes. We tell them that the doctors had told us that the patient was critically ill and that the patient needed to be shifted to the surgery or ICU just to check if there was any possibility. This makes the family members really understand the criticalness of the patient rather than having any false hopes.”—**TC7S** “Frankly speaking, we avoid using our transplant coordinator or transplant word. We introduce as a person who is there to explain them everything about the patient and that they can ask everything from us. Initially we try to avoid the word transplant coordinator… So, we play the role of a grief counsellor.”—**TC10N**

1. Supportive management policies

*Creating accountability and awareness of the team members’ role*: transplant coordinators described that increasing the sense of accountability from each of the team members by designating specific duties accompanied by the expectation of scrutiny improved performances, and mandatory reporting to the public will improve the efficiency of the donation process. A higher sense of such accountability and awareness of every team member’s role resulting in efficient processes were predominantly described to exist in private hospitals in both the regions and a few public hospitals in the south. In those centres, frequent meetings where they shared their experiences and lessons learnt made the team members realise their accountability and consider the importance their organisation give toward the organ donation and its process.

*Rapport, cooperation, and network*: effective rapport and cooperation between transplant coordinators and stakeholders, from senior consultants to housekeeping, was said to facilitate smooth deceased organ donation processes and improved performance. It was mentioned that public hospitals face this challenge more than private hospitals. However, good rapport, cooperation and network experiences were mostly described by participants in the south compared to the northern study sites. Also, majorly in the south, a common virtual chat group was described to enhance cooperation, enable real-time updates, make everyone prepared for their role and track progress. Additionally, building a network with transplant coordinators from various regions helped them to handle unfamiliar situations.

*Heavy workload:* though transplant coordinators find their role as a noble duty in being a means to save lives, in certain centres with less manpower, especially in public hospitals, they find this role heavily challenging leading to an ineffective deceased organ donation process, contrary to the situations in private hospitals. In some centres, doctors performing necessary tests like apnoea tests, declaring brain death, and attending late-night calls for deceased donation, were considered beyond their defined role due to the high patient load they already treat, especially in public hospitals in both the study site regions. In such cases, the question that arose was, why would doctors invest extra effort when time is scarce due to the existing heavy patient load and risking personal reputation in the complex and challenging brain dead organ donation process?

2. Infrastructure for the deceased organ donation process

*Lack of private space for discussion and counselling*: transplant coordinators found it challenging to have such sensitive conversations with the family members due to the lack of private and quiet spaces, which they find in private hospitals and very few public hospitals in the south. Such conversations, especially in public hospitals were identified to take place in corridors, making it uncomfortable, un-enabling to address their grief, poor concentration on the information being given, and high potential for intruders making it even more challenging. Majorly, private hospitals both in the north and south study sites were identified by the participants to have quiet and private spaces to address their grief, calm and ease them to have the conversation on deceased organ donation and enable them to ask their doubts for informed decision making, leading to effective decision making among the families.

*Lack of proper infrastructure in public hospitals*: the lack of well-equipped intensive care units (ICUs), insufficient medical instruments and devices, and well-trained teams were found to be other infrastructural barriers, especially in public hospitals both in the north and south of India.

3. Delays in processing time

*Delays related to medico-legal cases*: most of the deceased organ donations in India were reported to be from road traffic accident cases, which require police investigations due to medico-legal inquiries. The long distance between accident sites and hospitals was said to cause delays, as the local police officer from the site of the accident had to arrive at the hospital to conduct medico-legal inquiries. However, transplant coordinators only from Chennai (i.e., study site from south India) said that police officers in the hospital area are approved to initiate investigations until the other police officials from the accident site arrive resulting in improved efficiency and avoiding delays. Additionally, in Tamil Nadu (the name of the state where the southern region’s study site is located), approval for private hospitals to conduct post-mortems was also said to reduce delays. However, these initiatives were not identified in interviews from the north of India. Having more awareness sessions among the police officers in Chennai, Tamil Nadu has been said to positively help their supportive contribution and cooperation.

*Other complex team-related delays*: transplant coordinators informed that poor support and cooperation between the team members has caused delays in various stages of the deceased donation process. This included cases requiring night-time brain death declaration where four doctors must independently declare brain death twice at six-hour intervals, delay in blood investigation, and all such elements of the process. However, creating accountability and increased manpower has been said to address these delays in private hospitals.

4. Active involvement in the identification process

*Active role in potential donor identification*: in private hospitals, majorly in the south, frequent rounds in the emergency room and ICUs helped the transplant coordinators know what type of patients are admitted. This helped them to know the patient’s history and progress which aided them when they would turn into potential deceased organ donors. While having conversations with the family members, knowing the patient well beforehand helped them improve trust with the families.

*Understanding the family dynamic*: understanding the family dynamics ahead of time was also identified to aid the transplant coordinators in having such sensitive conversations, without hurting any of their feelings, when the time comes. Understanding their emotional status, identifying which stage of grieving they are in, their belief system, and their ability to understand medical conditions helped transplant coordinators to be prepared when the time came for a conversation on organ donation.

*Identifying the decision maker*: identifying the decision makers, the decision-making dynamics within the family, and the supportive and aggressive person, has helped in planning and smoothly carrying out the conversation without any avoidable emotional harm. However, in some situations, it has become difficult for the participants since the person who makes the decision in reality and the person who should give consent legally are different and have different views.

5. Explaining the concept of brain death

*Clash in accepting and understanding brain death*: both in private and public hospitals, understanding and accepting brain death as death by the families was challenging due to its contrast with conventional death signs, which is the declaration of death after cardiac arrest. Transplant coordinators said that because of this, in several instances, they struggled to get doctors to diagnose and declare brain death for the negative impact it could have on the doctors’ reputation among the public and to avoid unnecessary controversies, especially in public hospitals.

*Process of explaining*: among the interviews in the southern regions, to address such a concern, participants informed that doctors employ various methods to explain brain death to family members. Some orally describe it using scans, while others physically demonstrate it by temporarily removing ventilator support and illustrating changes like chest movements. However, transplant coordinators said that to effectively demonstrate that, families’ prior understanding of the basic concepts of brain death was crucial to prevent confusion, and enhance cooperation, especially when nerve reflexes can be mistaken for signs of life.

*Communication with families*: transplant coordinators informed that understanding family dynamics, and their stage of grief, helped transplant coordinators approach them effectively. Building rapport through regular updates about the patient’s situation and explaining deteriorations prepared families for potential outcomes, reducing shock. This gradual approach, with only factual information, and with no false hopes was said to aid families in progressing through grief stages, allowing them to make informed decisions on donations, and was said to prevent shock and aggression during the death declaration. Simplifying explanations, assuring autonomy in decisions, and giving enough time were said to be crucial components in guiding families through the process without coercion or hasty choices. This approach aided both private and public hospitals. However, this approach was a challenge in public hospitals, due to the lack of private spaces for such conversations, and enough time and attention to be given due to their heavy workload. Also, the designation of transplant coordinators was identified to be a challenge while introducing themselves to the families, where they preferred to introduce themselves as grief counsellors or social workers.

## Discussion

In the present study, in-depth interviews were conducted with experienced transplant coordinators in India to understand their experiences, barriers, and facilitators in the process of deceased organ donation and underpin what experiences in certain centres enable efficient performances resulting in higher donations compared to others within the same country. Overall, private hospitals had institutional-level practices that enabled efficient processes. Much of the supportive experiences were highly described by transplant coordinators in the south compared to the study sites from the north. This included creating supportive management policies and infrastructures, reducing delays, active identification processes, and approaches to explaining the concept of brain death.

As mentioned by Kute et al. (2020) on the need for ‘*well-nuanced counselling*’ to ‘*help facilitate greater acceptance of organ donation*’^5^ (pg. 39), the present study adds that creating accountability for each stakeholder, rapport building between the transplant coordinators and the team, and cooperative behaviours in the process of deceased organ donation enables ‘*well-nuanced counselling*’ and improved outcome. In 2016, a consensus conference took place in Ottawa, Canada, where experts came together and discussed improving organ donation by creating system accountability. A formal accountability framework to ensure that a missed organ donation opportunity is reported and investigated was recommended as an obligation to potential transplant recipients and their families^51^; but who should be accountable? In another study conducted in Canada, 59%, 44%, and 40% of the Canadian physicians proposed that the hospital, department/service, and individual physician respectively should be responsible if a referral of a potential donor was not made^52^. However, the present study strongly argues that the sense of accountability should not just be on the organisation or an individual but on every team member for their designated duties to be delivered appropriately and on time.

A social network analysis study showed that hospitals with essential elements like coordination structures, accountability, a sense of responsibility in everyone involved, and good team communication resulted in the optimal functioning of organ donation processes^53^. They are therefore not just applicable to certain countries or systems but even to India^54^. The present study showed accountability, good relationships, good rapport, cooperation, trust among the team members, supportive collaborations with police and legal officials, and understanding of the role of transplant coordinators in the process of deceased organ donation as key elements that led to efficient organ donation processes. If such a collaborative, accountable, and delegated approach is not identified in an organ donation centre as shown in the present study and other studies^55,56^, it will negatively impact the organ donation outcome and the transplant coordinators will then be overloaded leading them to burnout and thereby having an impact on their job pressure, compassion fatigue, well-being, and retention^22–24,57^.

Breaking bad news with families in a hospital setting has always been a challenging and devastating experience for healthcare professionals and families. A recent qualitative study in Australia explored how language and environment when receiving bad news can affect the patients and their families in such a situation^58^. Families who received bad news preferred to have it in a quiet space, with soft and empathic language, only true facts with no false hopes but with sensitiveness, with no rush but built it up slowly and preparing the family and patients before breaking the bad news rather than dropping it into a conversation, and in an emotionally safe environment. Transplant coordinators described creating such an environment helped in improving the consent rate and efficient organ donation process. Supporting this experience in India, a systematic review and another study demonstrated that a change in environment to a quieter and emotionally safe place increased the consent rate between 20 and 67%^59,60^. However, while private and quiet spaces have been used by various other countries^59,60^, this is not mandatorily fulfilled in all the organ donation centres in India, especially a challenge for the public funded hospitals.

Early identification in organ donation has been demonstrated as a facilitator in the organ donation process and in approaching families appropriately^10,53^. In the present study, accessibility to early identify potential donors and their families helped the transplant coordinators in learning the family dynamics which aided in planning their conversation well ahead of time, which was not uniform across the country.

Transplant coordinators are crucial in the organ donation process, they verify patients’ medical history and engage with families to secure consent while also providing grief counselling. To ensure a seamless process, transplant coordinators must possess both empathetic traits and comprehensive knowledge of organ donation procedures. Trainings given to the transplant coordinators in various aspects of the process such as legal, ethical, social, emotional, clinical, and other soft skills are often reported to enable them to perform well despite other challenges^54,61^.

To enhance the acceptance of brain death declarations where applicable and to improve the donor conversion rate from eligible to actual donors, training programs are being implemented for intensivists and anaesthetists. These programs aim to educate the medical community on the medical and legal aspects related to the declaration of brain death^5^. The National Medical Council in India also implemented new guidelines for the MBBS curriculum, which emphasise the need for comprehensive medical education, including organ donation and transplantation. As part of the Graduate Medical Education Regulations 2023, the curriculum aims to enhance the understanding of organ donation among medical students, thereby improving awareness and practices related to organ donation and transplantation^62^. Hence, the training needs should be focused on all the team players involved in the process of organ donation rather than transplant coordinator alone, which would then create a sense of accountability and knowledge among all team players to deliver their role’s duty.

Another challenge highlighted by transplant coordinators in this study was related to their job title. To alleviate confusion among grieving families, coordinators often chose to present themselves as grief counsellors, general counsellors, or social workers, rather than using the term “transplant coordinators” from the outset. This approach mirrors practices in the UK, where the role was initially termed Donor Transplant Coordinators (DTCs) but was rebranded as Specialist Nurses in Organ Donation (SNOD) in 2009 to better align with the needs of the families and streamline the donation process^63^. This re-branding was implemented to enhance the support provided to families during the donation process and streamline the overall procedure. The change aimed to address concerns about the terminology used during sensitive interactions with families, ensuring that the staff were seen more as healthcare professionals supporting the grieving process rather than just coordinators. Moving forward, it is essential for strategies in India to consider the societal context when determining suitable titles for transplant coordinators, as this could significantly improve the deceased donation process.

Almost every deceased organ donor in India is following a brain death. However, traditionally death is described as the cessation of heartbeat. This traditional, cultural and religious understanding of death has caused challenges in acceptance of brain death as death among the public in India^17^. The current study proposed various practices to make one understand the concept of brain death. Before making an organ donation request, making sure family members understand the concept of brain death was identified as critical^64^. Transplant coordinators informed that brain death was explained using scans and taking the families to the ICU. However, since family members may mistake spinal reflexes with life, transplant coordinators informed that it is also important to make sure that the family members understand the concept of brain death well before taking them to the intensive care unit (ICU). To help families understand spinal reflexes in brain-dead patients, they used familiar examples. They explained that when a deceased moves during cremation, it doesn’t mean the person is alive. Similarly, when a butcher prepares a chicken for processing by gently removing its head, any subsequent twitching does not signify that the chicken is alive. By relating these concepts, families were better prepared to see their loved ones in the ICU, making the experience less confusing and distressing, if they encounter any movements caused by spinal reflexes.

The interviews conducted revealed that organ donation practices in the southern regions of India demonstrate several key factors contributing to their efficiency. These include enhanced collaboration among team members, a strong sense of accountability, standardised protocols, and clearly defined roles for each participant in the process. Additionally, supportive infrastructure, as well as collaborative policies involving hospitals, the transportation sector, law enforcement, legal entities, and state government support, play a crucial role. By leveraging the successful practices from these well-performing centres and states, there is significant potential to replicate these models nationwide. Such an approach could elevate India’s standing as a leader not only in terms of the total number of organ donations and transplants^65^ but also in overall organ donation rate.

The study’s strengths lie in its adoption of an appropriate qualitative methodology, which enhances the credibility, authenticity, and trustworthiness of the findings by exploring the lived experiences of transplant coordinators^66,67^. Unlike quantitative research, which focuses on generalisability, qualitative research prioritises the richness of data obtained through theoretical saturation. In this study, in-depth interviews with transplant coordinators provide valuable insights into their experiences, contributing to a nuanced understanding of organ donation processes. However, a limitation of this methodology is its restricted generalisability; while the findings can inform future hypotheses, they may not be applicable across all contexts. Moreover, the study exclusively interviewed transplant coordinators, suggesting that further research involving other stakeholders could enrich the understanding of the phenomena. It is also important to note that while the recommended facilitators for improving organ donation can be implemented nationwide, regional variations may still exist^29^. These discrepancies could stem from differing levels of organ donation initiatives in various states, highlighting the need for targeted research to identify and address these gaps in practice.

## Conclusion

In conclusion, the systematic implementation of identified facilitators (Table 3) across organ donation centres in India holds substantial promise for enhancing organ donation outcomes nationwide. By drawing lessons from successful international models, India can adopt key strategies that include strengthening organisational structures, ensuring adequate training for dedicated healthcare professionals, fostering public engagement, and promoting culturally sensitive visibility^68–73^.

**Table 3:** Proposed recommendation framework for enhancing the efficiency of the deceased organ donation process for both private and public organ donation centres: insights from the current study.

Key areas	Recommendations
1. Sense of accountability	Create a sense of scrutiny among all the team players on their deliverables in the process of deceased organ donation.
	Mandate monthly structured meetings within each centre to update the larger clinical and non-clinical team including the hospital management team involved in the process of organ donation and transplantation.
	Mandate standardised, structured, and detailed reporting to the National Organ and Tissue Transplant Organisation (NOTTO) on various parameters/indicators, including but not limited to the number of potential deceased donors, number of potential deceased donors approached, number of approved and declined consent, time taken from identification to retrieval of organs where consent approved, reasons for delays, and other relevant and useful information.
	Increase visibility and awareness on the important role of every hospital staff on their cooperation toward organ donation processes.
5. Protocol for enabling teamwork	Standardised protocol should be implemented that informs the roles and responsibilities required and expected from each team player in the whole pathway/process of deceased organ donation from identification to the of handing over of the deceased donor to the families and follow-up after discharge clearly inform, as identified in well-performing centres in India.
	Should inform the role of transplant coordinators and strict cooperation of other team players in active donor identification and management.
7. Policies	Mandatory periodic standardised reporting to hospital management and National Organ and Tissue Transplant Organisation (NOTTO) with penalty/ strict actions if not adhered.
	MoU between the health sector and the police department for supportive policies as implemented in well-performing states.
	Collaborative work between the healthcare sector, the Ministry of Law and Justice and parliament to include definition of brain death as death in the Registration of Births and Deaths Act.
	Brain death is recognised as death in cases where the deceased’s family consents to organ donation. However, it should still be legally permissible to list brain death as the cause of death, even if the family declines consent for donation.
	Mandate brain death diagnosis and reporting to the state Authority who then should update the national authority as appropriate.
	Mandate a standardised uniform practice of brain death declaration and avoid inconsistencies.
	The designation of "*transplant coordinators*" should be considered for renaming, such as to "*grief counsellors*" as adopted by certain centres in India. Introducing as "*transplant coordinators*" during interactions with bereaved families, particularly at sensitive times increase the complexities of the request process and subsequent discussions. Renaming the role is intended to mitigate these challenges and facilitate more effective communication with families.
14. Infrastructure	Not just mandate the presence of transplant coordinators in organ donation and transplant centres but more importantly mandate a uniform enabling environment protocol for transplant coordinators to function efficiently.
	It should be mandated that every organ donation and transplant centre provide a dedicated private room for the use of transplant coordinators and family members. This room should offer an appropriate environment for sensitive discussions, such as communicating the diagnosis of brain death, and serve as a quiet, calm space for families to grieve. The room must ensure privacy and be free from interruptions to facilitate clear communication and address any concerns or doubts the family may have. Additionally, the room must be equipped with appropriate monitoring and surveillance systems to ensure the safety of both the families and the healthcare team.
	It is recommended to implement mandatory, high-quality training programs for all teams involved in the process of deceased organ donation, ensuring they are equipped with the necessary skills and knowledge to manage the process effectively and sensitively.
	It is recommended that intensive care units (ICUs) be well-equipped and staffed with clinically trained personnel who are proficient in the management of potential deceased organ donors. This ensures optimal care and facilitates the organ donation process.

Future research must focus on effectively converting public willingness regarding organ donation into actual behaviour concerning registration and consent, thereby adding depth to the understanding of the behavioural dynamics. Addressing accountability, improving teamwork, identifying process delays, and creating supportive infrastructure for sensitive discussions will be essential for advancing these efforts. Furthermore, collaboration with law enforcement can mitigate delays in medico-legal cases, while enhanced communication training for healthcare professionals will promote informed decision-making among bereaved families. By prioritising these areas through targeted policy initiatives and focused research, India can achieve self-sufficiency in deceased organ donation, ultimately enhancing health outcomes and saving lives across the country.

## Supplementary Information

Supplementary Information 1.

Supplementary Information 2.

## Data Availability

As per the ethics approval—All anonymised data is available upon request from the lead author (BPV).
