# Supplementary material for: A qualitative study exploring barriers and facilitators in deceased organ donation process among transplant coordinators in India
Source: Sci Rep. 2024 Nov 20;14:28773. doi: 10.1038/s41598-024-80290-9 (PMC11579380; doi:10.1038/s41598-024-80290-9)
Supplement: Supplementary file 2 — Supplementary Information 2. [file 41598_2024_80290_MOESM2_ESM.pdf]

Supplementary file 2

In-depth interview guide - Transplant coordinator

A. Ice-breaker

What do you feel about deceased organ donation? What are your views towards it?

B. Donor Identification

1. Can you take me through how a potential deceased donor is identified?
    a. What are your job responsibilities and involvement?
    b. Who are the other stakeholders involved and how are they involved?
    c. How do you feel other healthcare staff treat and consider this process and the relationship with you?
2. Can you explain how the diagnosis of brain death happens at your centre?
    a. How does this teamwork in this situation? What opinion do you have on the working ethics and commitment at this stage that is exhibited at your centre?
3. Who informs the family about this diagnosis and certification of Brain Death?
    a. How is it being conveyed at your centre?
    b. How do you feel it should be conveyed?

C. Counselling and Request

4. So, once you have a potential donor certified as brain dead, what do you do next?
    a. How is it done? Can you elaborate on your experience?
    b. Who will approach the eligible donor families and how?
    c. How is the topic of organ donation brought forth to them?
    d. Can you give me an example of the starting sentences that you would be using to begin this conversation and to introduce them to the concept of brain death and organ donation?
    e. How do you build a bond and relationship towards these families and make the discussion happen?

D. Donor Screening and Maintenance

5. How is the screening carried out at your centre?
    a. What role do you play? who is involved? and what role each of them play.
    b. Can you elaborate more?
    c. What are the difficulties that you experience in this process?

E. Medico-Legal clearance

6. How are MLC dealt with at your centre?
    a. What role do you play? who is involved and what role do each of them play?
    b. Can you elaborate more?
    c. What are the difficulties that you experience in this process?
7. Can you share with me your overall experiences with the MLC cases? This may include considering all your cases which were cooperative and non-cooperative.

F. Conclusion

8. Are there any other thoughts that you would like to share now that you feel you missed during this discussion? Is there anything else that you feel serves as a barrier or a facilitator in this process of making a deceased organ donation that you would like to mention now?
